# Prognostic Signature of Hepatocellular Carcinoma and Analysis of Immune Infiltration Based on m6A-Related lncRNAs

**DOI:** 10.3389/fonc.2021.691372

**Published:** 2021-08-30

**Authors:** Ting Guo, Kun He, Yifei Wang, Jingjing Sun, Yong Chen, Zelong Yang

**Affiliations:** ^1^Department of Obstetrics, West China Second University Hospital, Sichuan University, Chengdu, China; ^2^Department of Hepatobiliary Surgery, Xi Jing Hospital, Fourth Military Medical University, Xi’an, China

**Keywords:** hepatocellular carcinoma, long noncoding RNA, prognostic signature, immunotherapy, m6A

## Abstract

The relationship between m6A-related lncRNAs and prognosis in hepatocellular carcinoma (HCC) is not yet clear. We used Lasso regression to establish a prognostic signature based on m6A-related lncRNAs using a training set from TCGA, and then verified the signature efficacy in a test set. Fluorescence quantitative real-time PCR (qPCR), Survival analysis, clinical risk difference analysis, immune-related analysis, and drug-sensitivity analysis were conducted. The results revealed that 1,651 lncRNAs were differentially expressed in HCC tissues, among which, 163 were m6A-related. Univariate analysis showed that 87 lncRNAs were associated with the overall survival. Six differential m6A-related lncRNAs were validated and selected *via* Lasso regression to construct a prognostic signature which demonstrated a satisfactory predictive efficacy. In the clinically relevant pathologic stage, histologic grade, and T stage, the risk scores obtained based on this signature showed a statistically significant difference. The high- and low-risk groups exhibited a difference in the tumor immune infiltrating cells, immune checkpoint gene expression, and sensitivity to chemotherapy. In summary, the prognostic signature based on the m6A-related lncRNAs can effectively predict the prognosis of patients and might provide a new vista for the chemotherapy and immunotherapy of HCC.

## Introduction

Hepatocellular carcinoma (HCC) is the seventh most common form of cancer and the second most frequent cause of cancer-related mortality in the world. Its incidence is on the rise, posing a serious threat to human health (1). At present, there are multiple ways to treat HCC, such as partial hepatectomy, liver transplantation, radiofrequency ablation, hepatic artery embolization chemotherapy, and targeted therapy and immunotherapy. However, the efficacy of these therapies is limited by the high recurrence rates and high metastasis rates of HCC (2). Notably, the 5-year survival rate of HCC is only 18.4% in some developed countries although clinical technologies have advanced in recent years (3), and much lower in less developed countries because of access barriers to diagnosis and treatment. Due to the complex molecular mechanism of HCC and the heterogeneity of cancer cells, we urgently need a new and accurate method to better predict prognoses and develop personalized treatment plans.

N6-methyladenosine (m6A) is the most widespread form of post-transcriptional modification. The modified sequence is conservative and enriched near the termination codon, 3’untranslated region (UTR), and long introns and exons. The m6A modification can affect the expression of target genes, thereby impacting on the corresponding cellular processes and functions. Notably, m6A participates in almost all the steps of RNA metabolism, including mRNA translation, degradation, splicing, transport, and spatial folding. m6A-related enzymes can be divided into three types: writer, eraser, and reader, that play different roles in different tumors (4). For example, METTL14 suppresses the metastasis of HCC by modulating primary miR-126 processing (5). WTAP modifies ETS1 through m6A and then promotes the progression of HCC through the HUR1-ETS1-p21/p27 axis; WTAP can thus be used as an independent prognostic predictor of HCC (6).

Long noncoding RNA (lncRNA) is more than 200 nucleotides in length and does not encode any protein; it mainly plays a regulatory role. lncRNA dysregulation is involved in the pathological process of a variety of cancers, including cell growth and proliferation, drug resistance, and metastasis. For instance, the expression of lncRNA HULC is closely related to the tumor size and overall survival from HCC. It can act as a driver to prompt HCC proliferation, migration, and invasion *via* modulating the phosphorylation of YB-1 (7). In addition, lncRNAs such as MALAT1, HOTAIR, and H19 regulate the malignant biological behavior of HCC through ceRNA competition, regulating key oncogene transcription or other molecular mechanisms (8). Some specific lncRNAs, like lncRNA WRAP53, exist differently in the body fluids of HCC patients, which may serve as an independent prognostic indicator to predict the relapse of HCC (9).

The purpose of this study is to explore the differences in m6A-related lncRNAs of HCC and establish an effective prognostic signature. This signature is then used to stratify the HCC patients *via* risk scores. A signature-related prognostic analysis, immune infiltration analysis, immune checkpoint genes correlation, IC50 prediction of antitumor agents, enrichment analysis, and clinical correlation analysis are then carried out to verify the efficacy of the signature.

## Materials and Methods

### Data Source

The mRNA-sequencing data of HCC were obtained from the TCGA database portal (https://portal.gdc.cancer.gov/), which included 365 HCC samples and 50 adjacent samples. Clinical data for each patient were also obtained from TCGA. Inclusion criteria was a (1) histological HCC confirmation and (2) simultaneous available information on the mRNA expression profile data and prognosis. These 415 samples were used for differential gene analysis and m6A correlation analysis, and 365 HCC samples with clinical information were used when constructing the signature. We randomly selected 182 HCC cases as the training set, and the remaining cases as the test set.

### Differential Gene Analysis

We performed differential gene analysis between the tumor and control, and the gene differential expression was calculated using the “edgeR” package in R (10). We extracted all lncRNAs based on the gene type in the annotation file and conducted a differential analysis. The cut-off value for differential genes was the log fold change ∣logFC∣ ≥ 1, *p-value* < 0.05. All differential lncRNAs were selected for subsequent analysis.

### Correlation Analysis Between Differential lncRNAs and Key m6A-Related Genes

We obtained the list of the key m6A-related genes from a previous study (11). We conducted Pearson correlation analysis between the differential lncRNAs and m6A-related genes using the “psych” package in R. The screening criteria were correlation |R| > 0.5 and *p-value* < 0.05. The screened lncRNAs were further used for building the signature.

### Prognostic Signature Construction and Validation

We performed univariate Cox analysis to screen out lncRNAs related to the overall survival (OS) and included them in the Lasso regression. We used the “glmnet” package in R to build a Lasso regression to select genes included in the signature based on the training set and used COX regression to construct the signature and calculated the risk value corresponding to each sample (12). The cases were divided into high-risk and low-risk groups based on the median of the risk value. We constructed a time-longitudinal ROC curve based on different endpoints. We plotted a nomogram of the signature by using the “rms” package in R. Decision curve analysis (DCA) is used to calculate the clinical benefit of each model compared to all or none strategies (13). To assess the prognostic capacity of the six lncRNAs signature, DCA was conducted by using the “rmda” package in R.

### Fluorescence Quantitative Real-Time PCR and Cell Lines

MIHA (Human normal liver cell), LX-2 (Human normal stellate cell), and four human HCC cell lines, including huh7, HepG2, Li-7, and PLCPRF5, were all purchased from ATCC, and were cultured in DMEM (Gibco) supplements with 10% fetal bovine serum (Hyclone), 100 U/ml of penicillin (Gibco), and 0.1 mg/ml of streptomycin (Gibco) at 37°C in a humidified atmosphere of 95% O_2_, 5% CO_2_.

Total RNA was isolated using the TRIpure Reagent (RP1001, Bioteke). Reverse transcription was performed on 1 mg of RNA at 60°C for 35 min using a BeyoRT II M-MLV (D7160L, Beyotime). After reverse transcription, the cDNAs were used for semi-quantitative PCR by using 2×Taq PCR MasterMix (PC1150, Solarbio) and SYBR Green (SY1020, Solarbio). Amplification was carried out as recommended by the manufacturer in the qPCR instrument (Exicycler 96, BIONEER): 25 µl of reaction mixture contained 12.5 µl of SYBR Green mastermix, the appropriate primer concentration, and 1 µl of cDNA. The amplification program included the initial denaturation step at 95°C for 10 min, 40 cycles of denaturation at 95°C for 10 s, and annealing and extension at 60°C for 1 min. Fluorescence was measured at the end of each extension step. After amplification, melting curves were acquired and used to determine the specificity of the PCR products. 2 ^-△△CT^ method was used in data process.

The sets of specific primers as follows:

AC012313.8: 5’-CACCTGGAATCGGAAGT-3’
      5’-GGGAATGTGGCAGAAAG-3’;
AC092171.2: 5’-TGAGATACGGCGAGACCC-3’
      5’-GGACCGCTGTGCTGATGT-3’;
AL353708.1: 5’-TGTCGTCCAAATAAGTCG-3’
     5’-GTTAAAGCAAAGCCAATAC-3’;
KDM4A_AS1: 5’-ACATCTCATCTCGCCCTCC-3’
     5’-GTCTCCAGTTTGGTCCTCC-3’;
LINC01138: 5’-AATAGCGGCTGCTTCTTT-3’
     5’-TGGTCTGCATGGGATAGG-3’;
TMCC1_AS1: 5’-ATAAGGGAGGCAGAACGAGA-3’
     5’-GTCACAGGCCAGACTACCAG-3’;
β-actin: 5’-GGCACCCAGCACAATGAA-3’;
     5’-TAGAAGCATTTGCGGTGG-3’.

### Survival Analysis and Signature-Related Analysis

We used the “survival” package in R to perform a survival analysis and verified the conclusions in the test set. Only in the survival analysis, we divided the data into the training set and test set. In the rest of the various analysis related to signatures, our research object is the overall cases. To evaluate the correlation between the risk scores and clinical characteristics, we also compared the differences in the risk scores between the TNM stages, pathological stages, and histological grades, respectively. We conducted univariate and multivariate analysis to verify the independent predictive ability of the signature. We analyzed the differences in immune infiltration and immune checkpoint genes expression between the high- and low- risk groups, and we analyzed the correlation between each lncRNA in the signature and immune checkpoint genes. We chose LESCtin and PD-L1 as the research objects because these two genes are expressed on the tumor cell membrane, which will facilitate future experimental verification. The tumor immune dysfunction and exclusion (TIDE) value was calculated by online database (http://tide.dfci.harvard.edu/) (14), cancer type was set as “other”, previous immunotherapy was set as “none”. We made signature-related drug-sensitivity predictions using the “pRRophetic” package in R (15).

### Gene Set Enrichment Analysis

GSEA determines whether an *a priori* defined set of genes has statistically significant differences in expression under two different biological conditions (16). The GSEA was performed on the differential genes between the high-risk group and low-risk group by the GSEA software from Broad Institute (http://software.broadinstitute.org/gsea/downloads.jsp). A *p-value* < 0.05 was set as the cut-off criterion, and two databases had been used: GO and KEGG. The number of gene set permutations was 1,000 for each analysis.

### Statistical Methods

In the analysis of differences, we used Student’s t test to compare the means between the two groups and one-way ANOVA analysis to compare the means between multiple groups. We used Pearson correlation analysis to screen m6A-related lncRNAs and analysis the correlation between lncRNAs and immune checkpoint genes. In the process of constructing the signature, we used Lasso regression and COX proportional-hazards model. In the process of evaluating the signature, we used Kaplan–Meier survival analysis and log-rank test.

## Results

### Identification of Differential m6A-Related lncRNAs

**Figure 1:**
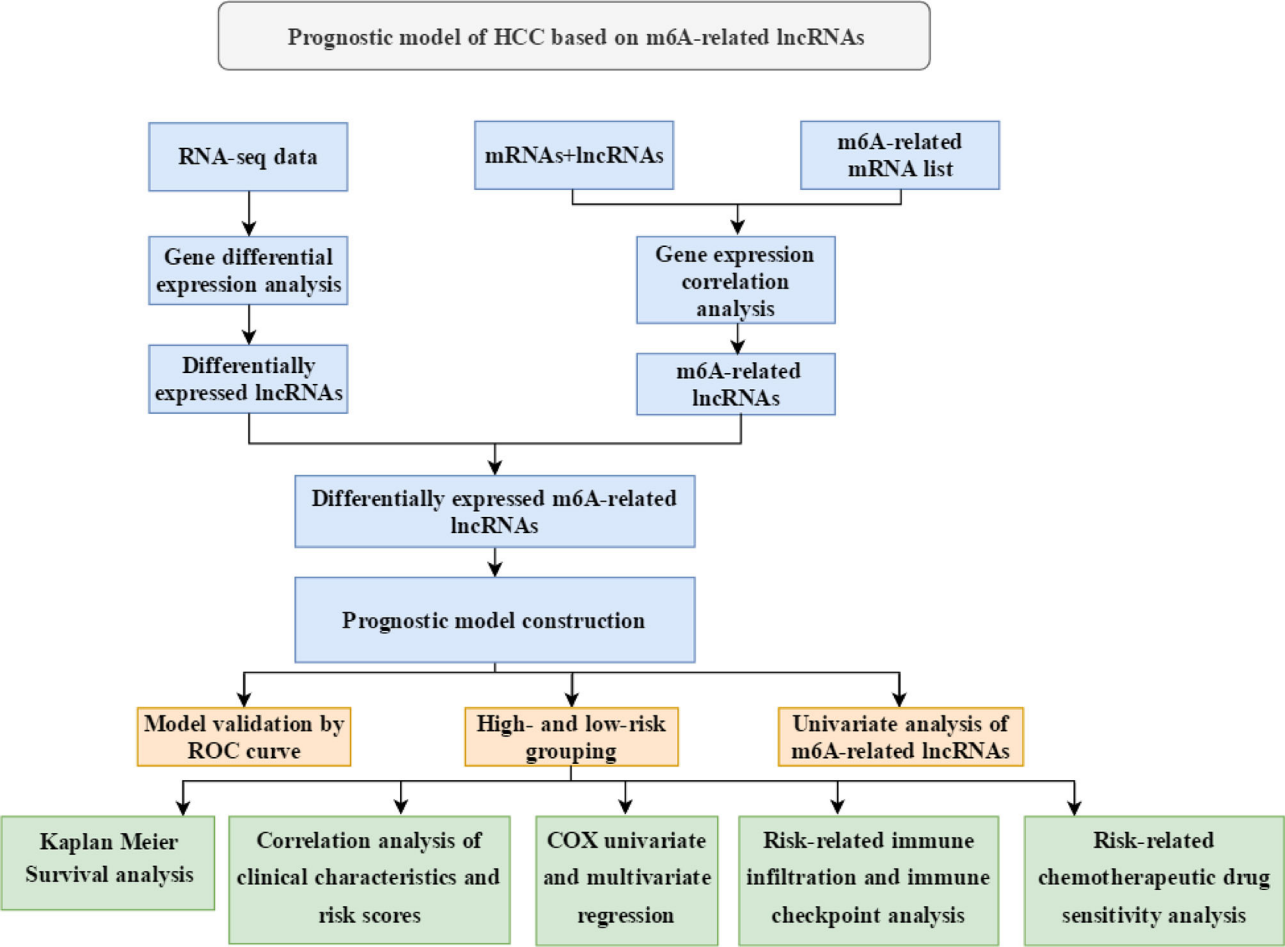
Technical roadmap in this study.

**Figure 2:**
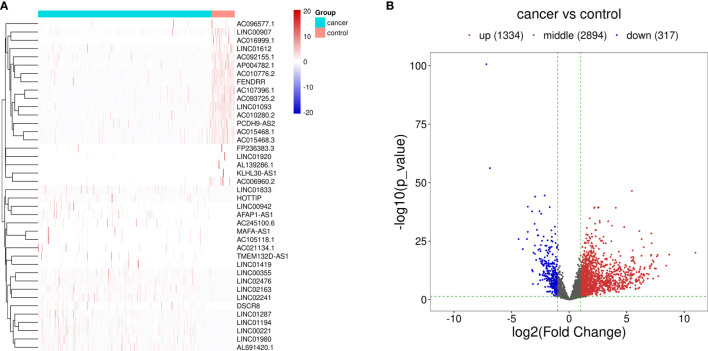
Differentially expressed lncRNAs between HCC cases and control cases. **(A)** Hierarchical clustering of the differentially expressed lncRNAs. The blue bar represents the cancer cases, and red bar represents the control cases. **(B)** Volcano map of the differentially expressed lncRNAs. Red dots represent the upregulated lncRNAs and blue dots represent the downregulated lncRNAs.

The procedure of this study is presented in **Figure 1**, and the clinical features of the patients included in the training and test sets are shown in **Table 1**. After the difference analysis, a total of 1,334 upregulated lncRNAs and 317 downregulated lncRNAs were identified. The representative results can be seen in **Table 2**. A heatmap of the top 20 differentially expressed lncRNAs is shown in **Figure 2A**. A volcano plot of differential expressed lncRNAs is shown in **Figure 2B**. We collected a total of 23 m6A-related key genes and conducted correlation analysis with differentially expressed lncRNAs. We screened out 163 positive m6A-related lncRNAs, the top 10 lncRNAs are shown in **Table 3**.

**Table 1:** Clinical characteristics of the included HCC patients from TCGA-HCC.

Variables	Total HCC (N = 365)	Training group (N = 182)	Testing group (N = 183)
**Age**	59.65 ± 13.36	61.25 ± 12.78	58.05 ± 13.76
**Status**			
Alive	235 (64.38)	120 (65.93)	115 (62.84)
Dead	130 (35.62)	62 (34.07)	68 (37.16)
**Sex**			
Male	246 (67.4)	123 (67.58)	123 (67.21)
Female	119 (32.6)	59 (32.42)	60 (32.79)
**T_Stage**			
T0	1 (0.27)	0 (0)	1 (0.55)
T1	180 (49.32)	87 (47.8)	93 (50.82)
T2	91 (24.93)	51 (28.02)	40 (21.86)
T3	78 (21.37)	37 (20.33)	41 (22.4)
T4	13 (3.56)	5 (2.75)	8 (4.37)
TX	1 (0.27)	1 (0.55)	0 (0)
Unknown	1 (0.27)	1 (0.55)	0 (0)
**N_Stage**			
N0	248 (67.95)	118 (64.84)	130 (71.04)
N1	4 (1.1)	2 (1.1)	2 (1.09)
NX	112 (30.68)	62 (34.07)	50 (27.32)
Unknown	1 (0.27)	0 (0)	1 (0.55)
**M_Stage**			
M0	263 (72.05)	129 (70.88)	134 (73.22)
M1	3 (0.82)	3 (1.65)	0 (0)
MX	99 (27.12)	50 (27.47)	49 (26.78)
**Pathologic_Stage**			
Stage I	170 (46.58)	81 (44.51)	89 (48.63)
Stage II	85 (23.29)	46 (25.27)	39 (21.31)
Stage III	83 (22.74)	39 (21.43)	44 (24.04)
Stage IV	5 (1.37)	4 (2.2)	1 (0.55)
Unknow	22 (6.03)	12 (6.59)	10 (5.46)

**Table 2:** The differentially expressed genes (Top 5).

Upregulated genes	log2FC	p-value	q-value	cancer	control
LINC01419	10.97	9.53E-21	3.76E-19	10.65	-0.32
LINC02241	8.69	6.27E-20	2.21E-18	7.69	-0.99
LINC01287	8.43	3.25E-15	4.74E-14	10.17	1.74
AC021134.1	7.74	1.07E-10	6.83E-10	6.66	-1.07
AC245100.6	7.68	7.48E-20	2.58E-18	7.4	-0.28
**Downregulated genes**					
KLHL30-AS1	-7.19	2.37E-101	1.07E-97	2.12	9.31
AL139286.1	-6.87	6.59E-57	1.49E-53	-1.1	5.77
AC107396.1	-4.39	1.40E-26	1.29E-24	2.11	6.5
AC006960.2	-4.02	2.62E-22	1.28E-20	1.4	5.42
AC093725.2	-3.66	1.31E-26	1.24E-24	0.26	3.93

**Table 3:** m6A related lncRNAs (Top 10).

mRNA	lncRNA	R	p-value
IGF2BP1	AC091133.4	0.729159139	0
HNRNPA2B1	AC091057.1	0.687591645	0
HNRNPA2B1	AC099850.3	0.684468872	0
RBM15B	BACE1-AS	0.678201483	0
METTL3	BACE1-AS	0.65054172	0
METTL3	TMEM147-AS1	0.640071009	0
RBM15B	SREBF2-AS1	0.639858788	0
METTL3	AC016394.1	0.636789277	0
METTL3	AC026362.1	0.636470469	0
YTHDC1	ZNF32-AS2	0.633987049	0

### Prognostic Signature Construction and Validation

**Figure 3:**
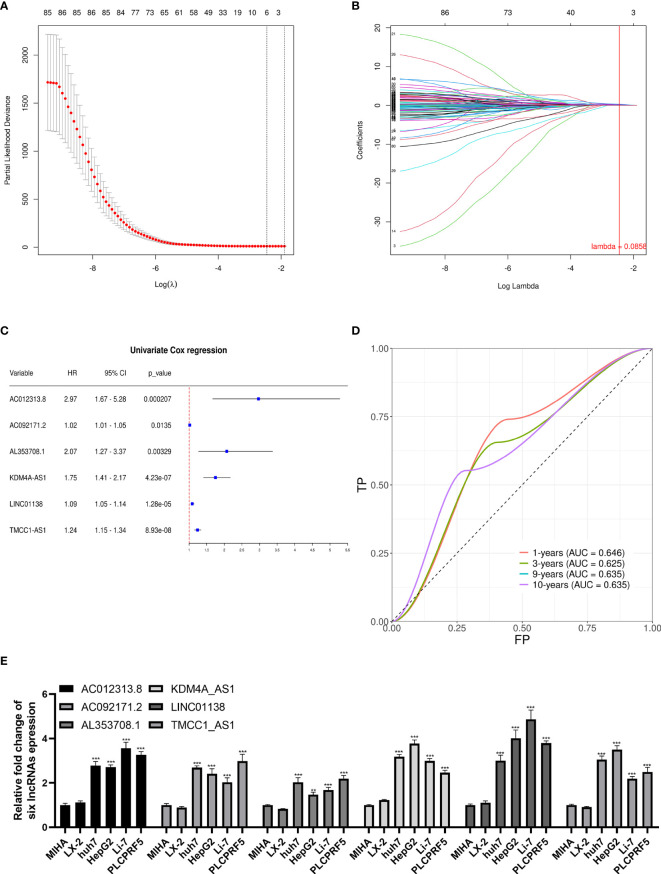
Lasso regression signature construction and validation. **(A)** Determination of the penalty value based on the lowest point of the lasso regression curve. **(B)** The lncRNAs that make up the signature were included according to whether the penalty values intersected each curve, and the regression coefficients were calculated. **(C)** Univariate cox regression analysis of the lncRNAs in the signature revealed that each lncRNA in the signature was associated with prognosis. **(D)** The calculation of the prediction accuracy of the signature at different time nodes using the ROC curve. **(E)** AC012313.8, AC092171.2, AL353708.1, KDM4A-AS1, LINC01138, and TMCC1-AS1 are significantly expressed higher in HCC cells than normal liver cells. **p < 0.01, ***p < 0.001.

We performed univariate Cox regression on the abovementioned m6A-related differential lncRNAs, and selected prognosis-related lncRNAs *via* Lasso regression to construct a prognostic signature by Cox regression. A total of six lncRNAs were included in the signature (**Figures 3A, B**). The signature is as follows:

risk score=0.318941965∗AL353708.1+0.199130468∗KDM4A_AS1+0.108210024∗TMCC1_AS1+0.107201455∗AC012313.8+0.014315484∗AC092171.2+0.006178981∗LINC01138.

The univariate Cox regression results of the lncRNAs in the signature are shown in **Figure 3C**. Each lncRNA in the signature is significantly related to a shorter overall survival of HCC patients. To verify the signature, we constructed the ROC curve with 1, 3, 9, and 10 years as the prediction endpoints (**Figure 3D**). The highest area under curve (AUC) is 0.646 and the lowest AUC is 0.625.

These six lncRNAs in the signature are more highly expressed in liver cancer cells than normal liver cells and hepatic stellate cell (**Figure 3E**).

### Prognostic Value of the Signature

**Figure 4:**
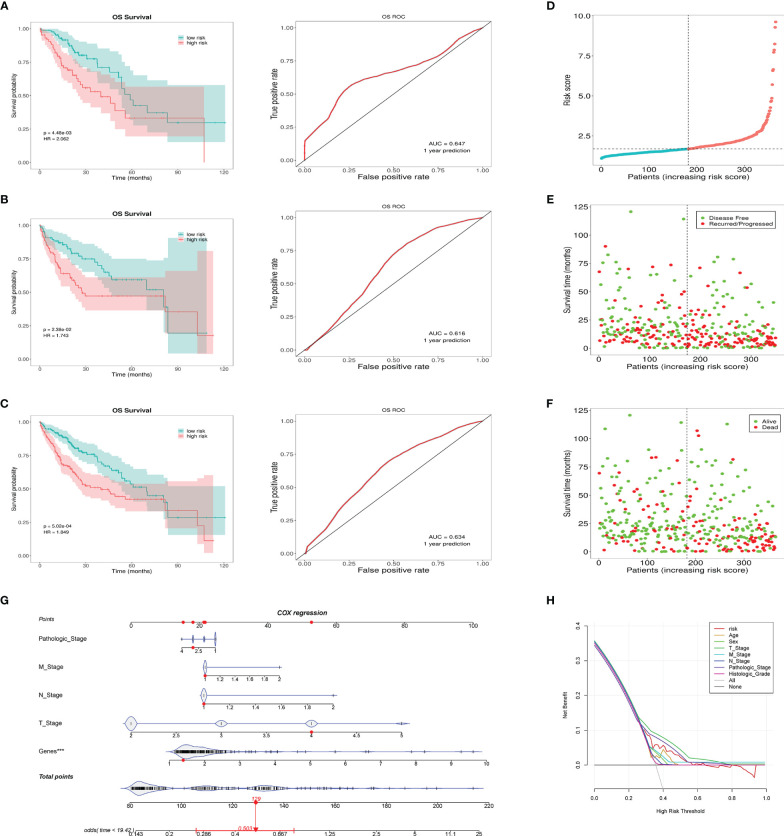
This prognostic signature predicts differences in prognosis between the high- and low-risk groups. **(A)** The survival curves of the high- and low-risk groups in the training set and the corresponding 1-year ROC curves. **(B)** The survival curves of the high- and low-risk groups in the testing set and the corresponding 1-year ROC curves. **(C)** The survival curves of the high- and low-risk groups in the total cases and the corresponding 1-year ROC curves. **(D)** Risk score distribution of patients in the high- and low-risk groups. **(E)** Distribution of the overall survival status among HCC patients with an increased risk score. **(F)** Distribution of recurrence patients among HCC patients with an increased risk score. **(G)** Nomogram predicting the OS for HCC patients. The short black line and the blue fiddle box represent the data distribution. Genes refers to the risk value calculated by the signature. The red dots indicate the example. The red line represents the confidence interval of OS of the example. **(H)** DCA curve shows the signature has clinical value, but it is not better than the T stage and pathologic stage. ***p < 0.001, means the risk score of the lncRNAs is an independent indicator.

According to the risk grouping information, combined with the overall survival (OS), we conducted Kaplan–Meier (KM) survival analysis in the training set and test set, see **Figures 4A**, **B**, respectively. The survival analysis after merging the two sets is presented in **Figure 4C**. The results show that people in the high-risk group have a worse prognosis. In addition, we took 1 year as the end of the prediction time and plotted the corresponding ROC curve for each set. The signature shows a modest predictive ability: AUC = 0.634. As the risk increases, the number of deaths of HCC patients or the number of recurrences increases (**Figures 4D–F**). DCA shows that the signature has a certain application value (**Figure 4H**). We added the pathological stage and TNM stage based on the DCA result to the nomogram, in order to achieve a better predictive performance (**Figure 4G**).

### Independent Prognostic Analysis, Signature-Related Clinical Analysis, and GSEA Analysis

**Figure 5:**
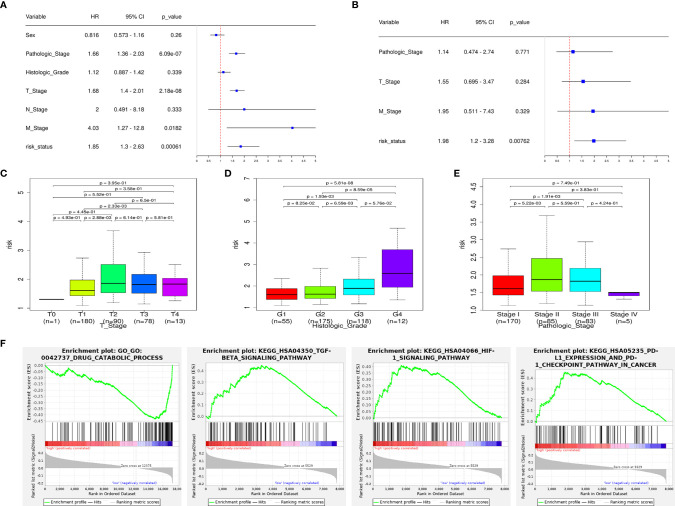
The signature has an independent predictive power and is closely related to the clinical parameters. **(A)** Univariate COX regression analysis shows that the pathologic stage, T stage, M stage, and risk status are all related to the overall survival. **(B)** Multivariate COX regression analysis shows that only the risk status obtained from the signature can be used as an independent prognostic factor. **(C)** Risk scores of patients in the T-stage differ by stage. **(D)** Risk scores of patients in the histologic-grade differ by grade. **(E)** Risk scores of patients in the pathologic-stage differ by stage. **(F)** GSEA results: the TGF-β signaling pathway, HIF-1 signaling pathway, PD-L1/PD1 checkpoint pathway are enriched in the high-risk group, drug catabolic process is enriched in the low-risk group.

Univariate Cox regression shows that the pathologic stage, T stage, M stage, and risk status are all related to the OS of HCC patients (**Figure 5A**). Multivariate Cox regression shows that only the risk status is significantly related to the prognosis (**Figure 5B**). The results show that the m6A-related lncRNA signature can be used as an independent prognostic predictor. We used the signature to score the patients, and we divided them according to the pathologic stage, histologic grade, and TNM stage. The risk differences were then compared. The results show that the risk scores between M0 stage and M1 stage, and N0 stage and N1 stage, does not have a significant difference, the risk scores of the T2 and T3 stages are generally higher than those of the T1 stage, and the risk score of pathological stage II patients is higher than those of pathological stage I. As the histologic grade increases, the risk score also increases significantly. These results are visualized in **Figures 5C–E**.

According to the GSEA enrichment analysis, the TGF-β signaling pathway, HIF-1 signaling pathway, PD-L1/PD1 checkpoint pathway are enriched in the high-risk group, and drug catabolic process is enriched in the low-risk group (**Figure 5F**). In addition, representative results are presented in **Supplementary Data 1**.

### Risk-Related Immune Infiltration Analysis and Risk-Related Immune Checkpoint Gene Expression Analysis

**Figure 6:**
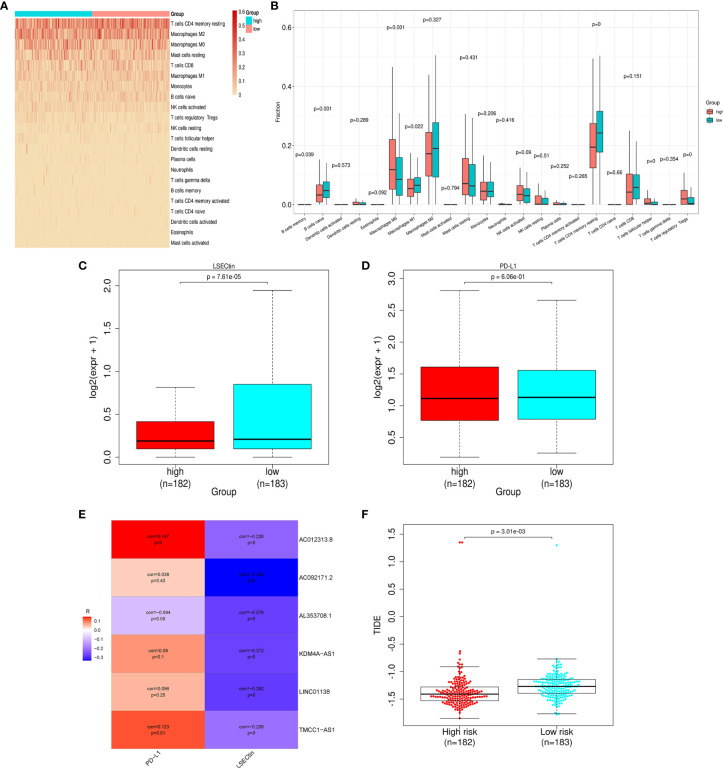
Differences in immune infiltration and immune checkpoint gene expression in the high- and low-risk groups. **(A)** Heat map of immune infiltration in the high- and low-risk groups. **(B)** Box plot of immune infiltration in the high- and low-risk groups. **(C)** Differential LSECtin expression in the high- and low-risk groups. **(D)** Differential PD-L1 expression in the high- and low-risk groups. **(E)** The correlation between each lncRNA in the signature and immune checkpoint genes. **(F)** The low-risk group has a higher TIDE score.

To explore the risk-related immune infiltration, we used CIBERSORT to score each sample (**Figure 6A**). Memory B cells, naive B cells, M1 macrophages, and CD4+ memory resting T cells have significantly higher scores in the low-risk group; T cells follicular helper, T cells regulatory Tregs, and M0 macrophages have significantly higher scores in the high-risk group (**Figure 6B**). In addition, we explored the differences in the expression of immune checkpoint genes in the high- and low-risk groups (**Figures 6C, D**). LSECtin is significantly higher in the low-risk group, suggesting that immunotherapy may be more effective in that group. But there was no significant difference in the PD-L1 expression between the groups. As for the correlation between each lncRNA in the signature and the above two immune checkpoint genes, only AC012313.8, TMCC1_AS1 are weakly correlated with PD-L1, and the others are insignificant. The lncRNAs in the signature are all negatively correlated with LSECtin (**Figure 6E**). The TIDE score was significantly higher in the low-risk group, indicating that patients in the low-risk group may get more clinical benefit from immunotherapy (**Figure 6F**).

### Risk-Related Drug-Sensitivity Prediction

**Figure 7:**
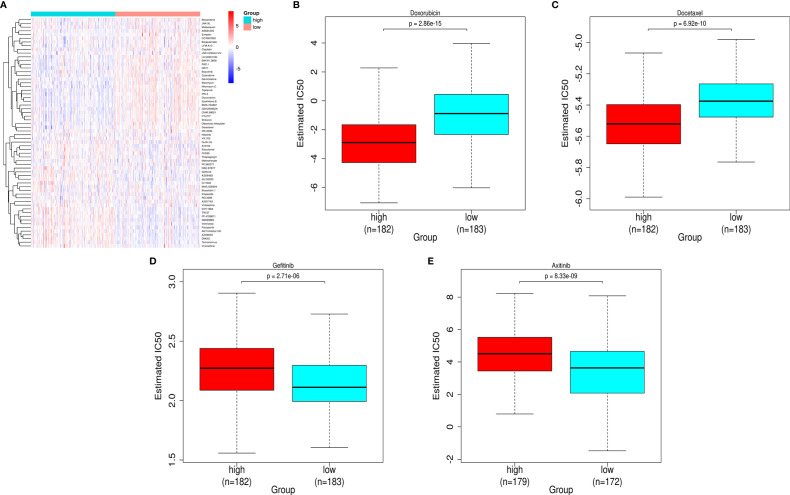
Prediction of the differences in HCC chemotherapy drug sensitivity in the high- and low-risk groups. **(A)** Heat map and clustering analysis of IC50 prediction of HCC chemotherapeutic agents in the high- and low-risk groups. **(B)** Differences in doxorubicin sensitivity in the high- and low-risk groups. **(C)** Differences in docetaxel sensitivity in the high- and low-risk groups. **(D)** Differences in Gefitinib sensitivity in the high- and low-risk groups. **(E)** Differences in Axitinib sensitivity in the high- and low-risk groups.

Mining the Cancer Genomic Project (CGP) database led to a total of 62 HCC-related drugs being obtained. We analyzed the IC50 of these drugs for cases in the high-and low-risk groups (**Figure 7A**). Among the drugs with differential sensitivity, we selected some representative and commonly used clinical drugs for display, like doxorubicin and docetaxel which may be more effective in the high-risk group as well as Axitinib and Gefitinib which may be more effective in the low-risk group (**Figures 7B–E**).

## Discussion

The prognosis of HCC remains unfavorable. m6A modification and numerous lncRNAs are closely related to the occurrence and development of HCC. Prior research has established effective signatures (17, 18), but, hitherto, no study has sought to construct a prognostic signature of m6A-related lncRNAs. Therefore, we integrated these two perspectives, exploring the predictive value of m6A modification-related lncRNAs in the prognosis of HCC. Given that m6A-related genes play an important role in the occurrence and development of HCC, we believe that the m6A-related lncRNAs are more likely to affect the prognosis of HCC and can form a more meaningful signature, so in our signature, only the m6A-related lncRNAs are retained for subsequent analysis.

In this study, we used the TCGA data to analyze the differences in lncRNA expression. There are 87 m6A-related prognostic lncRNAs that were included in the Lasso regression and a novel prognostic signature of six m6A-related lncRNAs was created. The six lncRNAs are AC012313.8, AC092171.2, AL353708.1, KDM4A-AS1, LINC01138, and TMCC1-AS1, and we verified that these six lncRNAs are highly expressed in HCC cell lines by qPCR. Among them, KDM4A-AS1 was included in the prognostic signature of HCC in another study (19). LINC01138 was reported to be an oncogenic driver that accelerates HCC cell proliferation, tumorigenicity, invasion, and metastasis, *via* interacting with arginine methyltransferase 5 to prevent its ubiquitin/proteasome-dependent degradation. LINC01138 itself can be modified and recognized by the m6A enzyme to maintain some oncogenes transcriptional stability (20). In addition, another study showed that LINC01138 could promote the lipid desaturation and cell proliferation of clear cell renal cell carcinoma *via* increasing arginine methylation and maintaining oncoprotein stability, which in turn affects patient prognosis (21). TMCC1-AS1 is included in several prognostic signatures of HCC as an autophagy-related lncRNA and its expression value is negatively correlated with the prognosis of HCC patients (22). After scoring each sample using the signature, the samples were grouped into high- and low-risk groups, and the prognostic differences between the groups were compared and verified in the validation set. We found that patients in the low-risk group had significantly longer survival times and better prognosis, and the ROC curve proves that the signature behaves satisfactorily in terms of predictive performance in the short term and the long term. Our signature has a higher efficacy and more function, like immunotherapy guidance, than some previously reported prognostic indicators (23, 24).

We found that as the histological grade increases, the risk score becomes higher, and the prognosis of the patient becomes worse, the relationship between histological grade and HCC prognosis is also consistent with previous reports (25). In the risk comparison of the M stage and N stage, the sample size of the M1 and N1 stages is too small, each stage group has only four cases, so the nonsignificant difference cannot form an effective conclusion. Similarly, in the T1 stage in the T staging and the IV stage in the pathological staging, there is a similar problem of an insufficient sample size. However, we clearly find that the risk scores of patients in T2 and subsequent stages are higher than those of patients in T1; tumor size is unequivocally related to the prognosis of HCC (26). Furthermore, the risk scores of patients in the pathologic stage II and subsequent stages are higher than those of patients in the pathologic stage I; the higher the pathologic stage, the worse the prognosis of HCC (27). Therefore, the differences in the risk scores between each T stage and between each pathological stage are partly objective and credible. Next, we incorporated the clinical factors and risk scores into univariate analysis and multivariate analysis, and found that only the risk status is significantly correlated with the overall survival. This indicated that our signature can independently predict the prognosis of HCC, and is not affected by confounding clinical factors. We performed functional enrichment and pathway enrichment analyses of the differential genes between the high- and low-risk groups, the differential gene enrichment of TGF-β, HIF-1, and PD-L1/PD1 checkpoint pathways in the two groups may give a biological explanation for the differences in the survival and clinical characteristics between the two groups.

Immune infiltration is closely related to the prognosis of patients with HCC (28). To highlight the research characteristics of this study and the immune relevance of the signature, the relationships between the risk score and tumor immune cell infiltration were investigated. We found that memory B cells, naive B cells, M1 macrophages, and CD4+ memory resting T cells were significantly increased in the low-risk group. It has previously been reported that these cells were increased in HCC tissues, and memory B cells, naive B cells, and M1 macrophages were associated with a superior survival (29). T cells follicular helper, T cells regulatory Tregs, and M0 macrophages were significantly increased in the high-risk group according to our results. Another study concluded that T cells follicular helper and M0 macrophages were unfavorable prognostic markers (30), which is corroborated herein. As is known, Tregs form an important part of the tumor immunosuppressive microenvironment. The number of local Tregs infiltrations is closely related to the progression of HCC, the more Tregs, the worse the prognosis (31). Therefore, we can conclude that our signature can appropriately distinguish partial types of immune infiltration cells in HCC and could potentially be leveraged to inform the development of immune cell-related therapies. Notably, LSECtin is found to be significantly higher in the low-risk group, which may indicate that patients in this group will respond better to immune checkpoint inhibitors. Although some studies have shown that the high expression of PD-L1 promotes tumor cells epithelial-mesenchymal transition and metastasis, leading to a poor prognosis (32), a meta-analysis showed that PD-L1 expression does not affect the prognosis (33), therefore, there is no difference in the expression of PD-L1 between the high- and low-risk groups, and it does not mean that the PD-1 immunotherapy have the same efficacy in the two groups. Based on the IC50 difference of various liver cancer-related drugs in the high- and low-risk groups, we can conclude that the signature we constructed can also be used to predict the sensitivity of chemotherapy drugs, and it could have a role to play in terms of guiding clinical chemotherapy.

This paper has some potential weaknesses that should be addressed. First, this study was mainly based on public databases, and the AUC of the signature is relatively less satisfied, this signature should be further optimized to improve its prediction accuracy and treatment efficiency, such as combine the signature with the TNM stage or another verified signature. Second, the physical and pathological functions of the six m6A-related lncRNAs in HCC require further exploration through a series of experiments. Third, validation of the efficacy of this signature was only undertaken at the data set level, and, in the future, it should also be performed on a large clinical scale. Despite the above shortcomings, our signature can effectively distinguish the prognosis of high- and low-risk patients and give positive suggestions on the clinical treatment. It reveals the research potential of the m6A modification and related lncRNAs in HCC to a certain extent.

## Conclusion

Our study identified a prognostic signature based on six m6A-related lncRNAs to predict the overall survival of patients with HCC. The risk scores were confirmed to be closely associated with the progression and immune infiltration of HCC. The IC50 of chemotherapy drugs can be predicted based on the signature, so the signature has some potential clinical significance. The prognostic signature could reliably predict the prognosis in HCC and may facilitate the development of individualized immune treatment plans.

## Data Availability Statement

The datasets presented in this study can be found in online repositories. The names of the repository/repositories and accession number(s) can be found in the article/**Supplementary Material**.

## Author Contributions

ZY and TG acquired and analyzed the data. YW, JS, and KH drafted the manuscript. YC and ZY are responsible for the integrity of the work as a whole. All authors contributed to the article and approved the submitted version.

## Funding

The present study was supported by the research funds from the National Natural Science Foundation of China (grant no. 8170563).

## Conflict of Interest

The authors declare that the research was conducted in the absence of any commercial or financial relationships that could be construed as a potential conflict of interest.

## Publisher’s Note

All claims expressed in this article are solely those of the authors and do not necessarily represent those of their affiliated organizations, or those of the publisher, the editors and the reviewers. Any product that may be evaluated in this article, or claim that may be made by its manufacturer, is not guaranteed or endorsed by the publisher.
